# “Caught in the Middle”: Factors Associated With Intention to Leave Among Long‐Term Care Managers in Atlantic Canada

**DOI:** 10.1155/jonm/7037794

**Published:** 2026-11-09

**Authors:** Kazeem Adefemi, Emily Rutledge, Lori E. Weeks, Kelli O’Brien, Debra Boudreau, Janice Keefe

**Affiliations:** ^1^ Faculty of Medicine Memorial University of Newfoundland St. John’s Canada mun.ca; ^2^ School of Health Administration Dalhousie University Halifax Canada dal.ca; ^3^ Catholic Health Alliance of Canada Ottawa Canada; ^4^ Nursing Homes of Nova Scotia Association Mill Cove Bedford Canada; ^5^ Department of Aging and Family Science and Nova Scotia Centre on Aging Mount Saint Vincent University Halifax Canada msvu.ca

## Abstract

**Aims:**

To investigate the factors associated with intention to leave (I2L) among long‐term care (LTC) home managers in Atlantic Canada, exploring the interplay between organizational context, burnout, and individual demographics using Herzberg’s two‐factor theory.

**Background:**

LTC managers face significant role strain as they are “caught in the middle” of competing systemic demands and frontline needs. Understanding the drivers of their turnover intention is critical for workforce stability, especially in regions facing rapid demographic aging.

**Methods:**

A cross‐sectional survey was completed by 89 managers (unit managers, directors of care, and facility administrators) across 53 LTC homes in Atlantic Canadian provinces. I2L was measured as a latent construct of withdrawal cognitions. Data were analyzed using generalized estimating equations (GEE) with a Gamma distribution to account for facility‐level clustering and skewed outcome variable.

**Results:**

Positive organizational culture (*B* = −0.25, *p* = 0.013) and high job satisfaction (*B* = −0.24, *p* < 0.001) were significantly associated with lower I2L. Conversely, higher burnout‐related cynicism significantly increased turnover intention (*B* = 0.07, *p* = 0.037). A potential “efficacy paradox” was observed, where managers with a higher sense of professional efficacy were more likely to report an intention to leave (*B* = 0.08, *p* = 0.039). While facility size and ownership were significant bivariate factors, they were not significant in the final model.

**Conclusions:**

Retention requires a dual approach that addresses both the context and content of managerial work. Organizations must improve “hygiene” factors by fostering a supportive culture to prevent cynicism. Simultaneously, they must provide “motivators” by ensuring managers have the autonomy and resources to do their jobs well.

**Implications for Nursing Management:**

Nursing leaders should move beyond individual resilience initiatives and prioritize modifiable organizational features. Potential strategies could include proactive measures to detect cynicism and the provision of structural authority to satisfy managers’ intrinsic need for professional achievement and impact.

## 1. Introduction

Long‐term care (LTC) is an integral pillar of Canada’s healthcare system [1]. In the Canadian context, LTC homes are funded by provincial governments and provide a range of health and personal care services to predominantly older adults who require 24 h nursing, personal, and support care [2]. This sector, in Canada, as well as globally, is experiencing rapidly increasing demand driven by an aging population and increasing complexity of care, necessitating a projected 100% growth in capacity over the next decade [3]. This urgency is especially true in Atlantic Canada, home to the country’s oldest populations, where over one in five adults are 65 and older [4]. While a robust LTC sector is critical to meeting this demand, worrisome workforce trends undermine the sector’s ability to respond effectively. Recent data indicate significant declines in the number of licensed practical nurses (LPNs) and registered nurses (RNs) working in the sector, which dropped by 6.1% and 2.1%, respectively, between 2021 and 2023 alone [3].

The LTC workforce faces unique challenges to their well‐being, including chronic stress, trauma, and significant occupational safety risks [5, 6]. The COVID‐19 pandemic further exacerbated these complexities, increasing the risk of burnout and “intention to leave” (I2L) among staff [7]. Within this context, I2L is understood through ‘Withdrawal Cognitions Theory’, which posits that the decision to leave an organization is a progressive psychological process rather than a singular event [8]. This process, often referred to as “withdrawal intention,” represents a latent construct encompassing a sequence of cognitive stages: thinking of quitting, the intention to search for alternatives, and the ultimate intention to quit [8–10]. Because these stages are highly correlated and reflect a singular underlying state of psychological detachment, I2L may serve as an important indicator of organizational stability and turnover risk [10].

Amid these workforce pressures, leadership has emerged as a critical factor in achieving the “Quadruple Aim” of healthcare—a framework dedicated to improving patient experience, enhancing population health, reducing costs, and, crucially, improving the work life of healthcare providers [11, 12]. LTC manager roles are central to this aim and the delivery of high‐quality, resident‐centered care. Their leadership capacity and the support they provide are fundamental drivers of organizational culture, staff wellbeing, and staff job satisfaction [13].

However, the COVID‐19 pandemic placed unprecedented strain on these leaders. Research indicates that during the COVID‐19 pandemic, managers experienced significant negative shifts in their work life, including sharp declines in job satisfaction and mental health alongside a marked increase in burnout [14]. While the acute phase of the pandemic has passed, the LTC sector has transitioned into a “new normal” characterized by chronic workforce instability and ongoing health system restructuring [7, 14]. Managers are now challenged with navigating the long‐term residue of pandemic‐related trauma while managing the regionalization and centralization of health authorities, which further contributes to role instability [7, 15].

On a regular basis, managers face a duality of stressors. Externally, they must navigate shifting government priorities, restructuring of health authorities, and stringent regulatory requirements [16]. In Canada, health authorities are regional or provincial organizations responsible for coordinating, regulating, and overseeing healthcare services, including LTC. Periodic restructuring can alter reporting relationships, accountability structures, and administrative priorities, creating additional uncertainty for LTC managers [15]. Internally, they manage high administrative workloads and the emotional burden of ensuring resident safety while mitigating staff burnout [16, 17]. Worrying about staff is a significant source of stress for managers which can have detrimental effects on their well‐being [14, 17]. This cumulative pressure positions LTC managers as being “caught in the middle,” balancing top‐down systemic demands with the bottom‐up needs of frontline staff and residents. Unlike frontline nurses and care aides, who primarily experience organizational policies as recipients, managers must simultaneously interpret, implement, and be accountable for those policies while supporting staff and maintaining quality resident care [16]. This persistent role strain significantly increases the risk of burnout and turnover among this critical leadership group [7]. While many of these pressures are shared by managers across healthcare settings, LTC managers navigate them within a sector that has historically received less policy attention, fewer resources, and lower professional visibility than acute care settings [18]. These sector‐specific challenges may intensify the impact of common managerial stressors and contribute to the unique workforce pressures observed in LTC.

Despite their central influence, empirical research examining the well‐being and I2L of LTC managers remains limited [19]. Recent findings from the Atlantic Research Collaboration on LTC found that collaborative team environment was the most important consideration and that managers more frequently prioritized general well‐being and management or leadership factors than the other occupational group [20]. Herzberg’s two‐factor theory offers a conceptual framework for examining turnover intentions among this workforce [21]. Herzberg described job satisfaction and dissatisfaction on two separate planes. Motivation factors, such as recognition and achievement, increase job satisfaction. Conversely, hygiene factors, such as policies and working conditions, prevent job dissatisfaction [22, 23]. Guided by Herzberg’s two‐factor theory, this study conceptualized organizational context variables (e.g., organizational culture, leadership, and social capital) as workplace “hygiene” factors that shape dissatisfaction and withdrawal. In contrast, individual factors relating to accomplishment and work meaning, particularly professional efficacy and job satisfaction, were conceptualized as reflecting motivational processes. Burnout dimensions were examined as potential mechanisms through which deficiencies in these hygiene and motivational conditions may contribute to turnover intentions.

However, little is known about the factors affecting I2L among managers, particularly in Atlantic Canada. Previous research suggests that structural characteristics of LTC management roles and care environments may influence workforce outcomes [24]. Differences in managerial role, facility size, and ownership model have been linked to variations in leadership responsibilities, organizational resources, job satisfaction, and turnover‐related outcomes among LTC managers and staff [24, 25]. Consequently, these factors were considered important contextual variables in the present study. Accordingly, this study has two primary aims: (1) to describe the LTC manager workforce in Atlantic Canada using demographic, quality of life, and organizational context indicators and (2) to determine which factors are most associated with managers’ I2L.

## 2. Materials and Methods

### 2.1. Study Design

The protocol for the study is outlined in Keefe et al. [2] as part of the Atlantic Research Collaboration on LTC (ARC‐LTC). This study used a cross‐sectional design with data collected from November 2023 to June 2024. The study was conducted across the four Atlantic provinces, including New Brunswick (NB), Newfoundland and Labrador (NL), Nova Scotia (NS), and Prince Edward Island (PEI). Manager surveys were self‐administered online, and facility‐ and unit‐level data were collected via personal interviews with Facility Administrators (FAs) or their designate.

### 2.2. Participants

Managers from eligible LTC homes were invited to participate in the study. A stratified random sample of LTC homes was used to select at least, 25% of homes in each of the four provinces. Where applicable, homes were stratified by size, ownership model, region, and language. NL and PEI homes were oversampled to ensure a minimum of eight homes were included from each province. Further details about stratification procedures have been published previously [2]. Managers were eligible if they had worked in their role for a minimum of 3 months and had direct oversight of staff and/or resident care. Managers self‐reported their role title as unit manager (UM), Director of Care (DoC), or FA.

### 2.3. Ethics

Ethics approval was obtained from multiple university ethics boards. Mount Saint Vincent University [2023‐039], University of New Brunswick [2023‐133], Memorial University of Newfoundland [2023.212], and Health Prince Edward Island. All participants were informed that by completing and submitting an online survey, they were providing consent for their information to be collected, analyzed, and included in any outputs derived from this research.

### 2.4. Measures

Variables were considered for this study based on prior literature on LTC workforce retention, Herzberg’s two‐factor theory, and data available through the ARC‐LTC study protocol. Demographic variables included age (< 50, ≥ 50), sex (male and female), place of birth (Canada, elsewhere), education (Diploma, Bachelors, Masters), province (NB, NL, NS, PEI), and years worked in current role and facility (continuous). Health and well‐being variables included the Short Form‐8 Health Survey (SF‐8) for self‐reported physical and mental health scores [26], the Maslach Burnout Inventory (MBI) for efficacy, cynicism, and exhaustion [27, 28], the Perceived Stress Scale (PSS‐4) for self‐reported stress [29], and the Michigan Organizational Assessment Questionnaire Job Satisfaction Subscale for job satisfaction scores (MOAQ‐JSS) [30]. Organizational context indicators were collected using the Alberta Context Tool (ACT) [31]. The ACT measures modifiable features of the work environment as perceived by the individual across 10 different domains including organizational culture, leadership, social capital, and organizational slack [31]. Consistent with Herzberg’s framework, organizational context indicators measured using the ACT were considered hygiene factors, whereas job satisfaction and professional efficacy were examined as indicators of motivational processes associated with achievement and professional accomplishment.

The primary outcome measure was I2L. This concept was measured via three five‐point Likert scale items. Items included “I often think about quitting,” “I will probably look for a new job next year,” and “I am currently looking for another job (in another organization).” I2L was measured using a composite score of the average of the three items. The variable demonstrated excellent internal consistency (Cronbach’s *α* = 0.95), confirming the items reliably measure a single construct [32]. Unidimensionality of the I2L scale was also confirmed through Principal Component Analysis (Eigenvalue = 2.6; 86.9% variance explained; all loadings > 0.9).

### 2.5. Analysis

For demographic, health, and well‐being variables, frequencies and percentages were calculated for categorical variables and mean and standard deviations for continuous variables. To manage the sample size (*n* = 89) and prevent model overfitting, a two‐stage modeling process was used. First, Spearman’s rank correlations were used to identify variables showing at least a moderate association (*r* > 0.25) with I2L for consideration in multivariable analyses. Spearman’s correlation was selected because several variables were ordinal and the I2L outcome demonstrated a non‐normal distribution (Schober et al., 2018). The *r* > 0.25 threshold was selected a priori as a pragmatic screening criterion to balance retention of potentially meaningful factors against the risk of overfitting given the available sample size. Variables considered during the screening stage included health indicators, burnout dimensions, job satisfaction, organizational context measures, and demographic characteristics (see supporting table S1).

For multivariable analysis, generalized estimating equations (GEE) model was used to account for potential clustering of managers within LTC homes [33]. Managers were nested within 47 LTC homes, with cluster sizes ranging from 1 to 10 managers per home (mean = 1.87; median = 1). An independent working correlation structure was specified, and robust (empirical/sandwich) standard errors were used for statistical inference. A Gamma distribution with a log link was selected because the I2L score was strictly positive and positively skewed. Diagnostic comparisons of residuals from Gamma and Gaussian specifications indicated improved residual behavior under the Gamma model [34, 35]. A parsimonious model was developed using a stepwise model‐building approach guided by the Quasi‐likelihood under the Independence Model Criterion (QIC and QICu), recommended for model comparison in GEE analyses [36]. Pairwise correlations and variance inflation factors (VIFs) were examined before model building to assess multicollinearity among candidate variables. No correlation exceeded 0.65, and all VIF values were below 5, suggesting that problematic multicollinearity was not present. Finally, a series of sensitivity analyses was conducted to evaluate the influence of structural variables (i.e., primary role, facility size, and ownership) on the final model. These variables were added individually to the parsimonious model to check for confounding and independent predictive value. In all checks, these variables were nonsignificant (*p* > 0.05). Consequently, they were excluded from the final model to maximize statistical power and maintain focus on modifiable organizational features.

Sample size was determined by the study sampling frame rather than an a priori power calculation. As part of the ARC‐LTC protocol, managers from a stratified random sample comprising at least 25% of LTC homes in each Atlantic province were invited to participate [2]. Of the 108 eligible managers, 89 completed the survey (82.4% response rate). The multivariable GEE analysis used complete‐case data from 84 managers, after five respondents with missing data on one or more variables required for the final model were excluded through listwise deletion. Because sample size was constrained by number of eligible managers within the study frame, we conducted a post hoc sensitivity analysis. With a complete‐case analytic sample of 84 managers, the study was adequately powered to detect moderate associations but was less well powered to detect smaller effects. Consequently, some weaker associations may not have reached statistical significance.

## 3. Results

### 3.1. Characteristics of the Sample

A total of 89 LTC managers across 53 LTC homes completed the survey, corresponding to an overall response rate of 82.4% among the eligible managers. After five respondents with missing data on one or more variables required for the final model were excluded through listwise deletion, the complete‐case GEE analysis included 84 managers clustered within 47 LTC homes. Most homes contributed one (55.3%) or two (27.7%) manager respondents. Detailed descriptive statistics for the sample are provided in Table 1. The participants were predominantly female (87%), and the vast majority were born in Canada (93%). Nearly half of the managers (47%) were aged 50 or older. The sample represented all four Atlantic provinces: NS (36%), NL (26%), NB (24%), and PEI (15%). Most of the managers (60%) had a bachelor’s degree and some (11%) held a Master’s degree as their highest educational qualification.

**TABLE 1:** Demographic characteristics of managers in Atlantic Canada LTC homes who completed the survey (*n* = 89).

Characteristics	*n*	%
*Age*		
< 40	19	21
40–50	28	32
≥ 50	42	47

*Sex*		
Male	11	12
Female	77	87

*Place of Birth*		
Born in Canada	83	93
Born elsewhere	6	7

*Role*		
Unit Manager	45	51
Director of Care	26	29
Facility Administrator	18	20

*Highest Education*		
Diploma/Certificate	25	28
Bachelor’s Degree	53	60
Master’s Degree	10	11

*Province*		
New Brunswick	21	24
Newfoundland and Labrador	23	26
Nova Scotia	32	36
Prince Edward Island	13	15

	**Mean**	**SD**

Years worked in current role	4.96	6.39
Number of years at current facility	8.21	10.20

The managers held various roles, with over half serving as UMs (51%), followed by DoC (29%) and FAs (20%). Regarding tenure, the sample had worked in their current role for an average of 5 years (SD = 6.39) and in their current facility for just over 8 years (SD = 10.2).

Well‐being and quality of work life (QWL) distributions are provided in Table 2. On a scale of 1–100, the mean physical health score was around the population average at 49.95 (SD = 7.74), while the mean mental health score was slightly below the population average at 47.18 (SD = 9.84). The average score for perceived stress was low at 4.75 (on a scale of 1–16). Managers reported high job satisfaction scores overall (mean = 4.21, on a 5‐point scale). While scores on the efficacy subscale of burnout were high (mean = 4.64), managers reported moderate scores on the cynicism (mean = 1.77) and emotional exhaustion subscales (mean = 2.12). Similarly, the reported mean score for I2L was moderate at 2.0 (SD = 0.94).

**TABLE 2:** Distribution of quality of work life and health of survey respondents.

	Mean	SD	Range	Possible range
*Health*				
Physical health	49.95	7.74	25.80–60.30	0–100
Mental health	47.18	9.84	14.50–61.80	0–100

*Quality of work life*				
MBI Emotional exhaustion	2.12	1.39	0–5.70	0–6
MBI Cynicism	1.77	1.34	0–5.30	0–6
MBI Efficacy	4.64	0.90	0–6.00	0–6
Perceived stress	4.75	2.83	0–13.00	0–16
Job satisfaction	4.21	0.63	2–5.00	1–5
Intention to leave	2.00	0.94	1–4.67	1–5

### 3.2. Bivariate Analyses

Preliminary bivariate analyses revealed significant structural differences in I2L (Table 3). At the role level, UMs reported higher I2L compared to FAs (mean: 2.27 vs 1.67; *B* = −0.307; *p* = 0.019). Significant differences were also observed based on facility size and ownership model. I2L was significantly higher in large facilities (120+ beds) compared to small facilities (mean: 2.48 vs 1.74; *B* = 0.354; *p* = 0.006). Also, managers in public facilities reported the highest I2L, and this was significantly higher than those in voluntary nonprofit facilities (mean: 2.49 vs 1.64; *B* = 0.418; *p* = 0.001).

**TABLE 3:** Bivariate analysis of primary role, size and ownership on managers’ intention to leave in Atlantic Canada LTC homes.

Variable	Categories	Mean (median)	Kruskal Wallis *X* ^2^ (*p*)	GEE B (*p*)
Primary Role	Unit Manager	2.27 (2.00)	5.70 (0.058)	ref
	Director of Care	1.77 (2.00)		−0.247 (0.082)
	Facility Admin	1.67 (1.50)		−0.307 (0.019)

Facility Size	Small	1.74 (2.00)	11.08 (0.004)	ref
	Medium	1.78 (1.50)		0.019 (0.869)
	Large	2.48 (2.30)		0.354 (0.006)

Facility Ownership	Public	2.49 (2.00)	14.12 (0.001)	0.418 (0.001)
	Private	2.03 (2.00)		0.213 (0.046)
	Voluntary	1.64 (1.33)		ref

### 3.3. Regression Model Findings

The final multivariable model identifying significant factors associated with I2L, after controlling for the included covariates and accounting for clustering of managers within homes, is presented in Table 4. Consistent with Herzberg’s conceptual framework, I2L was associated with both organizational (“hygiene”) and individual (“motivational”) factors.

**TABLE 4:** Factors associated with intention to leave among LTC managers in the final model (*n* = 84; 47 LTC homes).

Factors	Adjusted GEE coefficient, B (95% CI)	*p*
Intercept	2.15 (1.1709, 3.1300)	< 0.0001
MBI Exhaustion	0.04 (−0.0249,0.0951)	0.2517
MBI Cynicism	0.07 (0.0043, 0.1427)	0.0373
MBI Efficacy	0.08 (0.0039, 0.1581)	0.0396
Mental Health	0.00 (−0.0066, 0.0101)	0.6817
Job Satisfaction	−0.24 (−0.3727, −0.1063)	0.0004
Perceived Stress	0.01 (−0.0264, 0.0434)	0.6326
ACT Culture	−0.25 (−0.4394, −0.0525)	0.0127
ACT Leadership	0.01 (−0.0874, 0.1006)	0.8901
Province‐NB	−0.31 (−0.5030, −0.1167)	0.0017
Province‐NS	−0.13 (−0.3271, 0.0683)	0.1996
Province‐PEI	−0.21 (−0.4478, 0.0293)	0.0855
Province‐NL	ref.	
Age–50+	−0.14 (−0.2690, −0.0107)	0.0337
Age‐< 50	ref.	
Tenure‐5–10 years	0.02 (−0.1376, 0.1833)	0.7799
Tenure‐10+ years	−0.08 (−0.2506, 0.0941)	0.3734
< 5 years	ref.	

Among the organizational factors, managers who perceived a more positive organizational culture reported a significantly lower I2L (*B* = −0.25, *p* = 0.0127). Similarly, among the individual factors, job satisfaction was significantly associated with a lower I2L (*B* = −0.24, *p* < 0.001). In contrast, professional efficacy showed a significant positive association with I2L, with managers reporting higher self‐rated efficacy also reporting higher I2L in the final model (*B* = 0.08, *p* = 0.0396). This direction differed from the negative bivariate association between professional efficacy and I2L (*r* = −0.29), indicating that the association reversed after adjustment for the other factors in the model.

The other burnout dimensions showed contrasting effects. Higher levels of cynicism were associated with an increased I2L (*B* = 0.07, *p* = 0.0373), whereas emotional exhaustion was not a significant factor.

Demographic and provincial factors also remained significant, with managers aged over 50 (*B* = −0.14, *p* = 0.0337) and those in NB (*B* = −0.31, *p* = 0.0017) reporting lower I2L compared to those in NL. Other factors, including mental health status, perceived stress, and years in the role, were not significant factors in the final model.

## 4. Discussion

This study investigated the factors associated with I2L among managers of LTC homes in Atlantic Canada. Our findings support Herzberg’s proposition that workforce retention is shaped by both organizational conditions and intrinsic motivational processes. Significant factors were associated with I2L from both domains, with organizational culture representing an important hygiene factor and job satisfaction and professional efficacy representing motivational dimensions. Consistent with the literature on healthcare leadership retention [14, 25, 37], we found that a positive organizational culture and high job satisfaction were significantly associated with lower I2L among managers. Conversely, cynicism, a key dimension of burnout, and higher levels of professional efficacy were associated with higher I2L. Viewed through the lens of LTC management, these associations may reflect the challenges inherent in occupying an intermediary role between system‐level expectations and frontline care realities. Managers are responsible for translating policy, regulatory, and organizational priorities into day‐to‐day care delivery while simultaneously supporting staff and maintaining quality resident care [16, 18].

These findings are largely consistent with the broader nurse manager retention literature and align with findings from Dicks et al. [20], who used the same broader ARC‐LTC dataset to examine what managers, nurses, and allied health professionals identified as contributors to job satisfaction and found that managers cited ‘team dynamics’ among the most important factors contributing to their job satisfaction. Previous studies across LTC and other healthcare settings have similarly identified job satisfaction, organizational conditions, and burnout‐related factors as key predictors of turnover intention among nursing leaders [25, 37, 38]. One notable finding was the lower intention to leave reported by managers in NB compared with NL, suggesting that while the mechanisms associated with turnover appear broadly consistent across settings, their expression may be shaped by provincial policy and health system contexts [19]. The consistency of these findings across settings suggests that the organizational and motivational mechanisms influencing manager retention may be broadly transferable, even though their expression remains shaped by local policy and health system contexts. Consequently, retention strategies should be informed by both the wider evidence base and local system characteristics. The findings are also consistent with Herzberg’s Two‐Factor Theory and suggest that retention is affected by a dynamic interplay of ‘motivational’ and ‘hygiene’ factors [21].

We found that older managers (aged 50 and above) are significantly less likely to leave their positions. The observed association between older age and lower I2L is consistent with Perry et al. [39], who found that older nurses often find a better equilibrium in their roles and can be more focused on stability. However, the fact that 47% of our sample is over the age of 50 signals a looming “retirement cliff.” Short‐term stability among older managers may mask a long‐term crisis. If younger, highly efficacious managers are currently the most likely to leave, the sector faces a leadership vacuum. Cohen et al. [40] argue that retention strategies must be tailored to these unique demographic patterns rather than relying on general workforce trends.

The key factors associated with lower I2L in our study were high job satisfaction and a positive organizational culture. Managers reported a high mean job satisfaction score. This satisfaction showed strong inverse association with I2L. These findings align with Kennedy and Mohr [25], who found that satisfied nursing home workers had an 83% lower likelihood of reporting an intent to quit. Similarly, Penconek et al. [38] identified job satisfaction as the most consistent factor influencing the intent to stay across healthcare settings. Consistent with our findings, Aloisio et al. [24], in a study of 168 UMs, DoC, and FAs from Canadian residential LTC homes, found that organizational factors including social capital, leadership, and adequate orientation were significant predictors of manager job satisfaction.

These findings on job satisfaction and culture can also be understood through the lens of Herzberg’s Two‐Factor Theory. As Herzberg [23] theorized, organizational culture acts as a “hygiene factor,” representing the administrative and interpersonal context of the workplace. When this culture is positive, it prevents the dissatisfaction that drives managers away. However, the strong link between job satisfaction and retention suggests that managers also need “motivators.” These are intrinsic rewards like recognition and meaningful work [21]. As Wong et al. [41] noted, an authentic and supportive climate directly improves satisfaction. For Atlantic LTC managers, we can argue that a healthy workplace culture stops the “push” to leave, while high job satisfaction provides the “pull” to stay.

Another key finding in this study is that cynicism was significantly associated with higher I2L, while emotional exhaustion was not. Managers in our sample reported moderate levels of cynicism. This dimension of burnout appears to act as a bridge to turnover intention. Leiter and Maslach [42] argued that while exhaustion is about workload, cynicism is about “value conflicts” and/or “perceived unfairness.” For LTC managers these conflicts are intensified by being “caught in the middle”, constantly navigating conflicting pressures from frontline staff below, senior/board of directors above, and political or regulatory bodies externally. This unique structural position can lead to heightened psychological distress when managers feel unable to reconcile these competing demands. Leiter and Maslach [42] suggested that cynicism may act as a critical predictor of turnover, describing it as a form of psychological withdrawal.

While recent studies often focus on workload and staffing as primary drivers of burnout [7, 43, 44], our results suggest a more nuanced reality. LTC managers may be resilient to the physical exhaustion of a heavy workload but remain vulnerable to the perceived loss of value or meaning in their work. In Herzberg’s framework, cynicism is an expression of “hygiene failure,” occurring when perceived fairness, values, or organizational policies break down. This suggests that retention strategies must target the quality of the work relationship, rather than just the quantity of work [19]. Specifically, this refers to the quality of interpersonal relationships managers maintain with their staff and peers, alongside the necessary leadership capacity of managers to build and sustain these supportive relationships.

We observed an unexpected “efficacy paradox,” whereby managers reporting higher professional efficacy were more likely to report an I2L. This finding may appear counterintuitive, as professional efficacy is typically associated with engagement, competence, and positive workplace outcomes. For example, Aloisio et al. [37] found that efficacy was positively associated with job satisfaction among LTC managers. We suggest that a possible explanation is that managers with a strong sense of professional efficacy may be more inclined to consider leaving when organizational constraints, such as limited authority, staffing challenges, or insufficient resources limit their ability to exercise influence or implement meaningful change. [5]. This interpretation draws from the Herzberg’s framework, in which achievement and accomplishment function as key motivators [19, 21], and from qualitative findings reported by Estabrooks et al. [14], where managers described frustration arising from an inability to effect meaningful change. In this sense, professional efficacy may operate as a “frustrated motivator” and managers may consider leaving not because they feel incapable, but because they feel unable to achieve the level of impact they believe is possible within existing organizational conditions, similar to the “control paradox” described by Kennedy and Mohr [30]. However, this proposed mismatch between professional capacity and organizational influence was not directly measured and remains a hypothesis requiring further investigation in a larger study.

### 4.1. Implications for Nursing Management and Practice

Building on the factors associated with turnover intentions in this study, from the “caught in the middle” stressors to the efficacy paradox, we argue that LTC organizations and nursing leaders should focus on modifiable organizational elements to improve retention:

⁃ Organizations should move beyond “self‐care” initiatives and focus on the work environment. Improving leadership transparency, social capital, and organizational support are organizational “hygiene” factors that may help reduce cynicism and strengthen retention. This emphasis is supported by prior LTC research demonstrating that leadership, social capital, and adequate orientation are associated with higher manager job satisfaction and positive workplace experiences [24].
⁃ Retention strategies should focus on ensuring that managers have adequate decision latitude, access to resources, and opportunities to influence organizational change. While the positive association between professional efficacy and I2L warrants further investigation, it may suggest that managers with a strong sense of professional efficacy may be particularly sensitive to organizational constraints that limit their ability to make meaningful improvements. Providing managers with appropriate authority, administrative support, and mechanisms for participation in decision‐making may help reduce this potential mismatch between perceived professional capacity and organizational influence.
⁃ As younger managers reported higher I2L than managers aged over 50 in the final model, this suggests that mentorship, leadership development, succession planning, and peer‐support opportunities may be particularly relevant to this group. Although managers working in large and publicly operated facilities reported higher I2L in the bivariate analyses, these associations did not remain significant after accounting for other covariates. Future research should evaluate whether targeted supports improve retention‐related outcomes among younger managers and across different facility contexts.
⁃ LTC administrators should proactively monitor attitudinal shifts among managers. Early signs of cynicism, including perceived value conflicts and unfairness, may signal increased risk of psychological withdrawal and turnover intentions. Organizational assessment tools, such as the Areas of Worklife Survey, have been used to assess factors associated with burnout and workplace fit and may help organizations identify concerns before they become entrenched [45].

### 4.2. Limitations and Future Research

While this study provides valuable insights into an understudied area of the LTC workforce, several limitations must be noted. First, the final analytic sample (*n* = 84) was relatively small, which limited the number of variables included in the final model. Second, the cross‐sectional design precludes conclusions about causality. Third, the study measured self‐reported intention to leave rather than actual turnover; therefore, the findings should be interpreted in that context. The reliance on self‐reported measures may also be subject to social desirability or recall bias. Fourth, the variable‐selection process relied on bivariate screening followed by stepwise model building. Although this approach reduced the risk of model overfitting given the available sample size, it may have excluded variables with weaker independent associations and therefore should be interpreted as exploratory rather than definitive. Finally, while the focus on Atlantic Canada is a strength for regional policymaking, the findings may not be fully generalizable to LTC settings in contexts with different governance structures or funding models.

Future longitudinal research should examine whether and under what conditions managers’ intentions to leave translate into actual turnover in the LTC sector. Such research could also assess how changes in organizational culture and other workplace conditions are associated with I2L and retention over time. Qualitative investigations may also help to understand the mechanisms underlying the associations involving professional efficacy and cynicism with I2L. Lastly, comparative studies across Canadian provinces could help clarify the policy and health‐system factors that may contribute to provincial differences in I2L.

## 5. Conclusion

The factors associated with intention to leave among Atlantic Canadian LTC managers were largely consistent with those reported in the broader nurse manager retention literature. Job satisfaction and organizational culture emerged as key factors associated with lower I2L, while cynicism was associated with greater intention to leave. While these factors are broadly consistent with previous research, this study advances understanding of why they matter for LTC managers by interpreting them through two complementary lenses: Herzberg’s motivation‐hygiene framework and managers’ intermediary “caught in the middle” position between system‐level demands and frontline care realities. A notable exception was the positive association between professional efficacy and intention to leave, suggesting that highly capable managers may become dissatisfied when they lack the autonomy or resources needed to achieve meaningful change. Retaining LTC managers therefore requires a dual approach that addresses both the context and the content of the work. Organizations must strengthen organizational “hygiene” factors by fostering supportive workplace cultures that reduce cynicism while also providing the autonomy, authority, and resources needed to support managers’ professional achievement. Without this balance, managers may remain caught between organizational demands and frontline realities, threatening leadership stability and quality of care in LTC homes.

## Funding

This work was supported by the Canadian Institutes of Health Research [186055]; Research Nova Scotia [2022‐2407] and Research New Brunswick; Newfoundland and Labrador Centre for Applied Health Research; Health Prince Edward Island; and Nova Scotia Health.

## Conflicts of Interest

The authors declare no conflicts of interest.

## Supporting Information

Additional supporting information can be found online in the Supporting Information section.

## Supporting information


**Supporting Information 1** Supporting Table S1: Potential variables considered during model building, including screening correlations with intention to leave (I2L) and variance inflation factors (VIFs). This table provides the variables evaluated during the preliminary screening stage, their bivariate associations with I2L, and corresponding VIF values used to assess potential multicollinearity prior to multivariable model development.

**Supporting Information 2** STROBE‐Checklist.

## Data Availability

The data that support the findings of this study are available on request from the corresponding author. The data are not publicly available due to privacy or ethical restrictions.
